# The role of cytokines, adhesion molecules, and toll-like receptors in atherosclerosis progression: the effect of Atorvastatin

**DOI:** 10.25122/jml-2021-0187

**Published:** 2022-06

**Authors:** Ali Saud, Nabeel AJ Ali, Fadil Gali, Najah Hadi

**Affiliations:** 1 Department of Pharmacology and Therapeutics, College of Medicine, University of Kufa, Najaf, Iraq; 2 Department of Pharmacology, College of Medicine, University of Basra, Basra, Iraq

**Keywords:** atherosclerosis, toll-like receptors (TLRs), Atorvastatin, DMSO – Dimethylsulfoxide, HDL – High-density lipoprotein, ICAM – Intracellular adhesion molecule, IL-10 – Interleukin-10, IL17 – Interleukin-17, IL-1B – Interleukin-1β, LDL – Low-density lipoprotein, MCP-1 – Monocyte chemotactic protein, TG – Triglyceride, TLR2 – Toll-like receptor 2, TLR4 – Toll-like receptor 4, VCAM – Vascular cell adhesion molecule

## Abstract

Inflammatory cytokines, cell adhesion molecules, and toll-like receptors (TLRs) play an important role in atherosclerosis. The aim of this study was to further evaluate the role of inflammatory cytokines, cell adhesion molecules, and toll-like receptors in atherosclerosis. Forty local breed domestic male rabbits were divided randomly into 4 groups, 10 rabbits each. Group I was the control group, group II received a high cholesterol diet, group III received the drug solvent dimethyl sulfoxide (DMSO), and group IV received Atorvastatin (3.5 mg/kg/day). Blood samples were collected at 0 times, 5 weeks, and at the end of 10 weeks. TLRs expression on monocyte was measured by flow cytometry, IL-10, IL-17, IL-1β, intracellular adhesion molecule (ICAM), and vascular cell adhesion molecule (VCAM) were measured by ELISA. In group II, a high cholesterol diet led to a statistically significant elevation of lipids profile (TC, TG, and LDL) at both 5 weeks and 10 weeks compared to the control. The expression of TLRs was also increased compared to the control (13.53±2.5 to 25.79±6.5). The intimal thickness increased from 103.46±13.85 to 248.43±11.11. IL-17 increased significantly from 3.4±0.4 to 7.7±1.00, and IL-1β increased from 1.04±0.19 to 9.66±1.4 (P 0.05) at 10 weeks. ICAM and VCAM increased from 1.7±0.16 to 8.2±0.74 and from 0.89±0.07 to 5.2±0.45, respectively. Atorvastatin significantly reduced TLRs at 10 weeks to 21.98±3.4 and intimal thickness to 191.6±15.59. IL-17, IL-1β, ICAM, and VCAM were significantly reduced by Atorvastatin. Cytokines, cellular adhesion molecules, and probably TLRs have a role in the pathogenesis of hyperlipidemia and atherosclerosis.

## INTRODUCTION

Atherosclerosis is an inflammatory disease and a complex condition in which arteries become hard due to the formation of plaques. It is a multifocal and immune-inflammatory disease of large and medium-sized arteries [1]. It is characterized by vascular inflammation, endothelial dysfunction, and accumulation of lipids, cholesterol, calcium, and cellular debris inside the intima of the blood vessel wall (recruitment and activation of monocyte and macrophages and differentiation of vascular smooth muscle cells to form atherosclerotic plaque [2, 3]. Atherosclerosis is a multifactorial disease with different stages of inflammation from initiation to progression, and toll-like receptors (TLRs), notably TLR2 and TLR4, are involved in developing the atherosclerotic disease [4, 5]. Inflammatory cytokines are also involved in the initiation and progression of atherosclerotic changes. Furthermore, they are activated by TLRs stimulation during the inflammatory cascade involving adhesion molecules activation that initiates the problem [6]. Statins are commonly prescribed drugs in the treatment of atherosclerosis. Statins, in addition to lipid-lowering effects, can exhibit anti-inflammatory effects and modifications of TLRs [7].

The objective of our study was to evaluate further the role of TLRs as:

- Cytokines (IL-10, IL-17 and IL-1β), intracellular and vascular;
- Adhesion molecules (ICAM and VCAM) in the pathogenesis of atherosclerosis and to explore the modulator effect of Atorvastatin on these parameters.

## MATERIAL AND METHODS

Forty local domestic male rabbits were used in this study. Their weight ranged from 1–1.5 kg, and their age was between 6–12 months. These were housed in the same experimental conditions with constant humidity, and they were allowed to drink tap water and pellet diet *ad libitum*. After one week of adaption, the animals were randomized into four groups, ten rabbits each: normal control group (Ι) were kept on a standard chew diet and tap water, group (ΙΙ): atherogenic high cholesterol diet, were kept on an atherogenic diet (normal rabbit chew plus 2% cholesterol (wt/wt) [8] and tap water. Group (ΙΙΙ) was treated with dimethyl sulfoxide-DMSO and consisted of 10 rabbits kept on an atherogenic diet and tap water. DMSO (1ml) was given by oral gavages after dilution to a sufficient volume with distilled water. Finally, group (ΙV): treated with Atorvastatin and kept on an atherogenic diet. Atorvastatin was given (3.5 mg/kg/day) [9] by oral gavage. Atorvastatin tablets as calcium salt (Lipitor^@^). Atorvastatin was dispersed in DMSO since it is insoluble in water. About 3 ml of blood was collected from the central ear vein of each rabbit following overnight fasting. Blood samples were collected at zero time, at the end of 5 weeks, and the end of 10 weeks of drug treatment for each group of rabbits. Sera were collected to measure serum parameters. In the 10^th^ week, the animals were sacrificed under ketamine and xylazine (5 to 10 mg/kg) anesthesia. A segment of the aorta was dissected to estimate intimal thickness and histopathological examination.

### Lipid profile measurement

Plasma cholesterol, low-density lipoprotein (LDL), high-density lipoprotein cholesterol (HDL-C), and triglycerides (TG) were determined enzymatically by standard methods.

Measurements of TLRs were done by flow cytometry.

### Inflammatory biomarkers

An enzyme-linked immunosorbent assay (ELISA) kit was used for IL-10, IL-17, IL-1ß, ICAM, and VCAM serum levels measurement.

### Aorta intimal medial thickness

Measurements of intimal thickness were carried out by an ocular microscope.

### Histopathological procedure

Aorta segments were fixed in 10% formaldehyde at room temperature. The sections were stained with hematoxylin-eosin.

### Statistical analysis

Data are expressed as mean±SD. Paired t-tests were used to compare differences between the mean values within each group at different times. The Chi-square test was also used to compare histopathological findings in different groups. Statistical significance was considered as P<0.05. Analyses were performed using SPSS software update version 21.

## RESULTS

**Figure 1:**
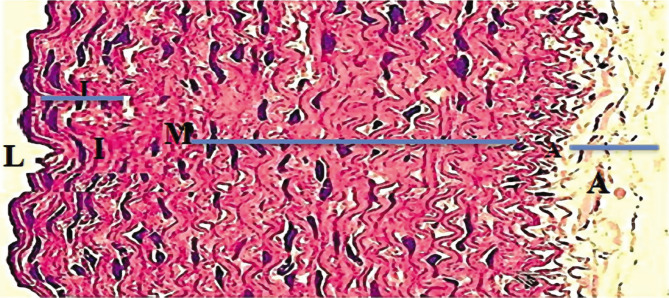
Histological section of rabbit aorta in the control group, normal layers represented by L – Lumen; I – intima layer; M – media layer; and A – adventitia layer (H and E 400X).

**Figure 2:**
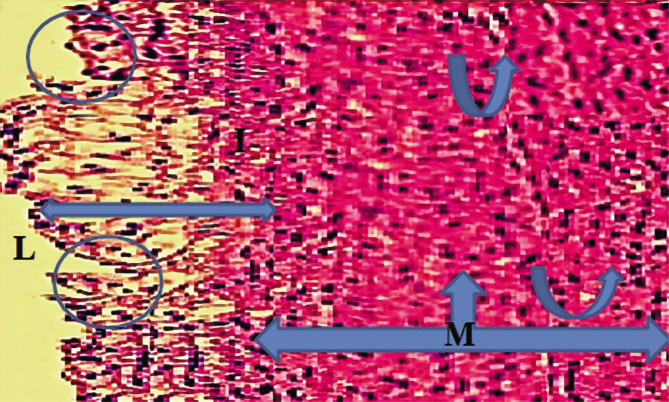
High cholesterol diet group hypercholesterolemia rabbits. L – Lumen; M – media layer.

**Figure 3:**
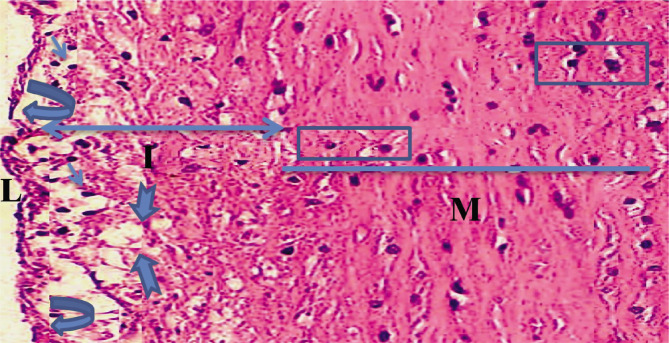
Atorvastatin groups aorta of rabbit represented by (straight M) media layer, (thick arrow) fatty streak and, (two head arrow I) intima layer, (square) fiber vacillation, (Arc arrow) necrosis of endothelium layer (H and E 400X) and (L) Lumen.

Atherogenic diet (group II) led to a clear and statistically significant (P<0.05) elevation of all the parameters of lipid profile (cholesterol, TG, LDL) (Table 1). The effect appeared at both 5 weeks and 10 weeks following ingestion with no significant effect on HDL. IL-17 significantly (P<0.05) increased from 3.4±0.4 to 7.7±1, and IL-1β from 1.04±0.19 to 9.66±1.4 at 10 weeks (Table 2). ICAM and VCAM significantly (P<0.05) increased from 1.7±0.15 to 8.2±0.74 and from 0.89±0.07 to 5.2±0.6, respectively. The expression of TLRs increased in comparison to the control group value (25.10±5.0 *vs*. 12.66±3.8 and 25.79±79 *vs*. 13.53±2.5), and the intimal thickness at 10 weeks significantly increased (P<0.05) from 103.46±13.85 in the control group to 248.43±11.11 in the atherogenic diet group. Atorvastatin treatment in group (IV) produced a statistically significant (P<0.05) reduction in lipid profile at both 5 and 10 weeks following treatment, with the largest effect at 10 weeks as compared to the high cholesterol diet group (group II), reduction of total cholesterol from 1301±443 to 290±23 and TG from 256.0±24.0 to 101.0±28 and LDL from 929±251 to 296±38 at 10 weeks. Atorvastatin also reduced serum level of IL-1β1 from 9.66±1.4 group (II) to 2.44±0.49 at 10 weeks, with similar changes in IL-17 and effect on IL-10. Atorvastatin also reduced both ICAM from 8.2±0.74 in group II to 2.7±0.13 and VCAM from 5.2±0.45 to 1.6±0.14 TLRs decrees from 25.79±6.5 to 21.98±3.4. This reduction was not statistically significant (P>0.05). Intimal thickness significantly decreased in the Atorvastatin group at 10 weeks from 248.43±11.11 to 191.6±10.59. In this study, all rabbits (100%) fed with high cholesterol diet developed hypercholesterolemia and atherosclerosis lesions with different stages in the branches of the aorta artery, including fatty streaks, atheroma, and fibrous cap formation. Treatment of rabbits with Atorvastatin resulted in a significant reduction in the severity of atherosclerotic lesions compared with the untreated group (Figures 1, 2, and 3).

**Table 1:** Changes of various atherosclerotic parameters in the four experimental groups.

Group		Zero week	5 weeks	10 weeks
Group I Control	TC mg/dl	59.86±15.16	61.56±19.00	61.19±14
	TG mg/dl	48.48±15.85	47±2.0	46±15.0
	LDL mg/dl	29±14.50	28±11.0	28.0±11.0
	HDL mg/dl	16.0±1.2	15.1.5±3.4	16.0±1.5
	TLR count	-	12.66±3.80	13.53±2.50
	IT µm	103.46±13.85		
Group II Atherogenic diet	TC mg/dl	63.94±16.15	717.64±209*	1301±443*
	TG mg/dl	42.05±3.51	196.4±45.35*	256.0±24.0*
	LDL mg/dl	30.0±11.0	576.0±190*	929±251.0*
	HDL mg/dl	18.0±4.2	16.0±3.5	16.0±2.1
	TLR count		25.10±5.0*	25.79±6.50*
	IT µm	248.43*±11.11		
Group III DMSO	TC mg/dl	63.52±13.17	785.98±271.00*	1136±371*
	TG mg/dl	47.25±16.68	187.4±26.15*	239.0±24*
	LDL mg/dl	24.0±11	460.0±75.0*	756.0±129.0*
	HDL mg/dl	18.0±3.2	17.0±2.5	15.0±3.0
	TLR count	-	21.49±3.8*	25.08±4.9
	IT µm	214.17*±12.89		
Group VI Atorvastatin	TC mg/dl	57.91±14.50	562.00±71*	290±23^α^*
	TG mg/dl	43.99±15.7	187.0±946.2*	101.0±28*^α^
	LDL mg/dl	38.0±13.0	502.0±67*	296.0±38*^α^
	HDL mg/dl	16.0±1.8	17.0±.5	22.0±2.0*
	TLR count	-	24.96±2.30*	21.98±3.40*
	IT µm	191.60*±10.593		

P<0.05 considered to be significant in comparison with the normal control group *. αP<0.05 considered to be significant in comparison with the atherogenic group. IT – Intimal thickness. Data are expressed as mean±sd (N=10 in each group).

**Table 2:** The effect of various treatments on inflammatory parameters.

Parameter	Group	0 time	5 weeks	10 weeks
IL-10 Pg/ml	I	2.10±0.10	2.13±0.08	2.02±0.07
	II	2.10±0.10	1.70±0.16	1.30±0.10
	II	2.15±0.10	1.70±0.12	1.29±0.10
	IV	2.17±0.09	1.7±0.07	1.9±0.08
IL-17 Pg/ml	I	3.55±0.40	3.45±0.24	3.40±0.22
	II	3.40±0.40	6.0±0.79	7.70±1.00*
	III	3.40±0.40	6.0±0.78	7.60±0.85*
	IV	3.40±0.18	6.10±0.31	4±0.15
IL-1β Pg/ml	I	0.99±0.1	0.95±0.09	1.03±0.08
	II	1.04±0.19	5.5±0.9	9.66±1.4*
	III	0.99±0.15	5.2±0.8	9.48±1.4*
	IV	0.99±0.2	5.25±1.0	2.44±0.49
ICAM Pg/ml	I	1.70±0.15	1.7±0.12	1.8±0.12
	II	1.70±0.16	4.2±0.44*	8.20±0.74*
	III	1.80±0.14	4.4±0.32*	8.20±0.6*
	IV	1.70±0.06	4.3±0.16*	2.70±0.13*^α^
VCAM Pg/ml	I	0.86±0.09	0.88±0.08	0.90±0.06
	II	0.89±0.07	3.0±0.27*	5.20±0.45*
	III	0.88±0.05	3.0±0.14*	5.30±0.30*
	IV	0.92±0.06	4.0±0.23*	1.60±0.14*^α^

P<0.05 considered to be significant in comparison with the normal control group *. αP<0.05 considered to be significant in comparison with the atherogenic group. Group I: normal control; group II: atherogenic diet; group III: DMSO; group IV: Atorvastatin.

## DISCUSSION

The high cholesterol diet in this study produced a clear and significant (P<0.05) increase in all the lipid profile parameters in rabbits. This is in agreement with many other studies [10]. It is known that rabbits rapidly developed hypercholesterolemia, with serum cholesterol of more than 1000 mg/dl following oral cholesterol feeding. Atherosclerotic changes in the form of foam cells occur within 8 weeks. A longer treatment period is not advisable as it is associated with failure to thrive and liver toxicity [11]. Rabbits are more sensitive to dietary cholesterol because they cannot increase sterol excretion, which increases LDL [12]. In this study, hypercholesterolemia resulted in an increase of IL-1β, and IL-17, with little effect on IL-10. IL-1β is a pleiotropic cytokine involved in the pathogenesis of atherosclerosis as it is a potent inducer of ICAM and VCAM expression in vascular smooth muscles [13]. IL-17 can also promote the adherence of monocytes to the vascular endothelial cells and stimulate the oxidized LDL [14]. On the other hand, IL-10 is considered an anti-inflammatory cytokine and was found to favorably influence the progression of atherosclerosis [15]. A high cholesterol diet in the present study also significantly (P<0.05) increased ICAM and VCAM; both are immunoglobulin-like adhesion molecules [16]. They mediate the interaction between endothelial cells and blood cells, an important mechanism of atherosclerosis progression [17]. Recently it was found that VCAM is mainly up-regulated following a high cholesterol diet [18]. Hypercholesterolemia can result in the augmentation of TLRs signaling, promoting inflammatory responses [19]. There is growing evidence of the contribution of TLR to the initiation and progression of atherosclerosis [20]. The expression of TLR2 and TLR4 at the blood vessel wall can enhance atherosclerosis in a synergistic way [20, 21]. This is likely due to marked augmentation of NF-κB, leading to augmentation of VCAM-1, ICAM-1, and MCP-1 (monocyte chemotactic protein) at the wall of the blood vessels [22]. Histopathological changes are in line with other results [23], which examined the rabbits using high-resolution magnetic resonance imaging (MRI) and found that the thickness of the vessel wall significantly increased (P<0.05) in cholesterol-fed rabbits. Elevation in the intimal thickness may be due to cholesterol crystal deposition. In our study, hypercholesterolemia was associated with an inflammatory response due to elevation in plasma levels of pro-inflammatory cytokines. There is evidence that inflammation can be involved in many processes like endothelial dysfunction, expression of many adhesion molecules, and alteration in smooth muscle cell function [24], which act as a procoagulatory, prothrombotic, and pro atherosclerosis environment [25].

This study found that 30% of rabbits treated with Atorvastatin for 10 weeks developed initial lesions, 50% developed intermediate lesions, and 20% developed advanced lesions. It was found that Atorvastatin can reduce inflammatory cell accumulations and smooth muscle cell proliferation and migrations, and plaque formation [26]. There is evidence that plaque stabilization is parallel with a decrease in lipid content and smooth muscle migration [27, 28]. Atorvastatin produced a statistically significant reduction (P<0.05) in serum lipids, reducing the intimal thickness and histopathological changes of atherosclerosis. The hypolipidemic effect of statins due to lipid synthesis inhibition is well known [23]. In addition, statins have many pleiotropic effects; however, the mechanism of such effects is not well understood. Atorvastatin had no significant (P>0.05) effect on TLR in the present study.

## CONCLUSION

In conclusion, a high cholesterol diet induced elevation of all serum lipid parameters. It also led to increased serum levels of IL-17 and IL-1β and increased adhesion molecules ICAM and VCAM. In addition to its lipid-lowering effect, Atorvastatin can also modulate cytokines and adhesion molecules, which could explain some of its pleiotropic effects. The role of TLR in the mediation of statins action requires further exploration.

## Data Availability

Data analyzed in this article is available from the first author.
